# Tripartite motif containing 28 (TRIM28) promotes breast cancer metastasis by stabilizing TWIST1 protein

**DOI:** 10.1038/srep29822

**Published:** 2016-07-14

**Authors:** Chunli Wei, Jingliang Cheng, Boxv Zhou, Li Zhu, Md. Asaduzzaman Khan, Tao He, Sufang Zhou, Jian He, Xiaoling Lu, Hanchun Chen, Dianzheng Zhang, Yongxiang Zhao, Junjiang Fu

**Affiliations:** 1 Key Laboratory of Epigenetics and Oncology, the Research Center for Preclinical Medicine, Southwest Medical University, Luzhou, Sichuan 646000, China; 2 State Key Laboratory of Quality Research in Chinese Medicine, Macau University of Science and Technology, Macau (SAR), 999078, China; 3 National Center for International Research of Biological Targeting Diagnosis and Therapy & Guangxi Key Laboratory of Biological Targeting Diagnosis and Therapy Research & Collaborative Innovation Center for Targeting Tumor Diagnosis and Therapy, Guangxi Medical University, Nanning, Guangxi 530021, China; 4 Department of Biochemistry, School of Life Sciences & the State Key Laboratory of Medical Genetics, Central South University, Changsha, Hunan 410013, China; 5 Department of Bio-Medical Sciences, Philadelphia College of Osteopathic Medicine, Philadelphia, Pennsylvania 19131, USA

## Abstract

TRIM28 regulates its target genes at both transcriptional and posttranscriptional levels. Here we report that a TRIM28-TWIST1-EMT axis exists in breast cancer cells and TRIM28 promotes breast cancer metastasis by stabilizing TWIST1 and subsequently enhancing EMT. We find that TRIM28 is highly expressed in both cancer cell lines and advanced breast cancer tissues, and the levels of TRIM28 and TWIST1 are positively correlated with the aggressiveness of breast carcinomas. Overexpression and depletion of TRIM28 up- and down-regulates the protein, but not the mRNA levels of TWIST1, respectively, suggesting that TRIM28 upregulates TWIST1 post-transcriptionally. Overexpression of TRIM28 in breast cancer cell line promotes cell migration and invasion. Knockdown of TRIM28 reduces the protein level of TWIST1 with concurrent upregulation of E-cadherin and downregulation of N-cadherin and consequently inhibits cell migration and invasion. Furthermore, Immunoprecipitation and GST pull-down assays demonstrated that TRIM28 interacts with TWIST1 directly and this interaction is presumed to protect TWIST1 from degradation. Our study revealed a novel mechanism in breast cancer cells that TRIM28 enhances metastasis by stabilizing TWIST1, suggesting that targeting TRIM28 could be an efficacious strategy in breast cancer treatment.

Transcription factor TWIST1 plays important roles in different processes of cancer development including metastasis, generation of cancer cell stemness and drug resistance, and these characteristics make TWIST1 an oncoprotein^1^,^2^,^3^. Mechanistically, TWIST1 enhances metastasis by promoting epithelial-to-mesenchymal transition (EMT)^4^,^5^,^6^,^7^. Loss of cell-cell adhesion and polarity, down-regulation of epithelial markers, and acquisition of mesenchymal markers/phenotype are all characteristic hallmarks of EMT^6^,^7^,^8^. In addition, altered expression of TWIST1 and/or hyper-methylation of its promoter regions have been implicated in the development of different cancers, including breast cancer^1^,^8^,^9^.

TWIST1 is a basic helix-loop-helix (bHLH) protein encoded by *TWIST1* gene, located on human chromosome 7p21^10^. TWIST1 has been considered as a master regulator of EMTs, and TWIST1 mutation is associated with autosomal dominant Saethre–Chotzen syndrome^11^,^12^. However, whether TWIST1 plays any role in breast cancer development is unclear because breast cancer risk in patients with Saethre-Chotzen syndrome is unchanged^13^. Down-regulation of cell-cell adhesion molecules such as E-Cadherin and up-regulation of more plastic mesenchymal proteins such as N-Cadherin are the major mechanism of TWIST1 action. However TWIST1 is also involved in cancer metastatic processes through other signaling pathways^1^,^2^,^3^,^8^. Since TWIST1 plays pivot roles in carcinogenesis, targeting TWIST1 has become an interesting research area for target-specific cancer therapeutics^3^. Consequently, identification of factors responsible for overexpression and modification of TWIST1 as well as its promoter hyper-methylation has also attracted more attention recently.

The tripartite motif-containing 28 (TRIM28) protein, also known as KRAB domain-associated protein 1 (KAP1) or transcriptional intermediary factor 1 beta (TIF1β), serves as a transcriptional co-repressor by interacting with a large family of KRAB-containing zinc finger protein (KRAB-ZFP) transcription factors^14^,^15^. TRIM28 regulates gene expression by affecting the transcriptional activity of KRAB-ZFP-specific loci, trans-repression or epigenetic modulation of chromatin structure. In addition, TRIM28 may also function as a transcriptional elongation factor because it is capable of binding to the promoter regions of transcriptionally inactive genes, permitting Pol II accumulation and making these genes ready for transcription upon induction^16^,^17^. Moreover, TRIM28 can also serve as a SUMO/ubiquitin E3 ligase^15^,^18^,^19^ and regulate apoptosis independent of its transcriptional activities. It has also been reported that by recruiting HDAC1 to MDM2-p53 complex, TRIM28 acts cooperatively with MDM2, an ubiquitin E3 ligase, to induce p53 degradation^20^,^21^. Therefore, TRIM28 may be able to promote neoplastic transformation by suppressing p53-mediated apoptosis. Recently, it has been reported that TRIM28 is capable of promoting breast cancer cell proliferation and metastatic progression^22^. Interestingly, TRIM28 is involved in the fibroblast-specific protein 1 (FSP-1)- or TGF-β-induced EMT in lung cancer^23^,^24^. These findings together indicate that TRIM28 may be involved in EMT and EMT-associated proteins and transcript factors (EMT-TFs), such as E-cadherin, N-cadherin, TWIST1, and SNAIL1. In this study, we demonstrated that TRIM28 interacts with and stabilizes TWIST1. Based on the role of TRIM28 in EMT, we propose that in breast cancer cells exist a TRIM28-TWIST1-EMT axis which plays an important role in breast cancer metastasis.

## Results

### TRIM28 is highly expressed in metastatic cancer cell lines and positively correlated with the levels of TWIST1 in advanced breast cancer tissues

**Figure 1:**
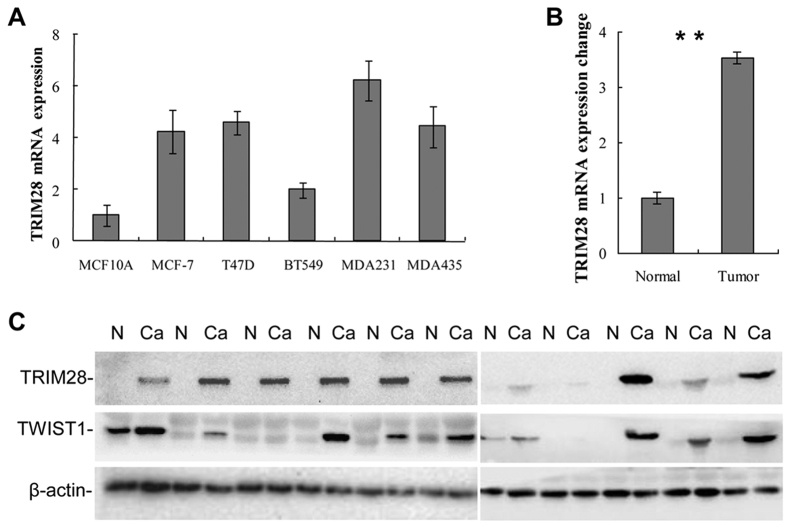
TRIM28 expression in different cell lines and invasive ductal breast cancer tissues. (**A**) TRIM28 mRNA expression in different cell lines by qPCR. (**B**) TRIM28 mRNA expression by qPCR in different invasive ductal breast cancer tissues and normal adjacent tissues. **P-value ≤ 0.05. (**C**) Representative Western blotting for TWIST1 and TRIM28 protein expression in eleven invasive ductal breast cancer tissues and matched adjacent normal tissues. TWIST1 protein expression was positively correlated with TRIM28 protein expression in the breast tumors.

**Figure 2:**
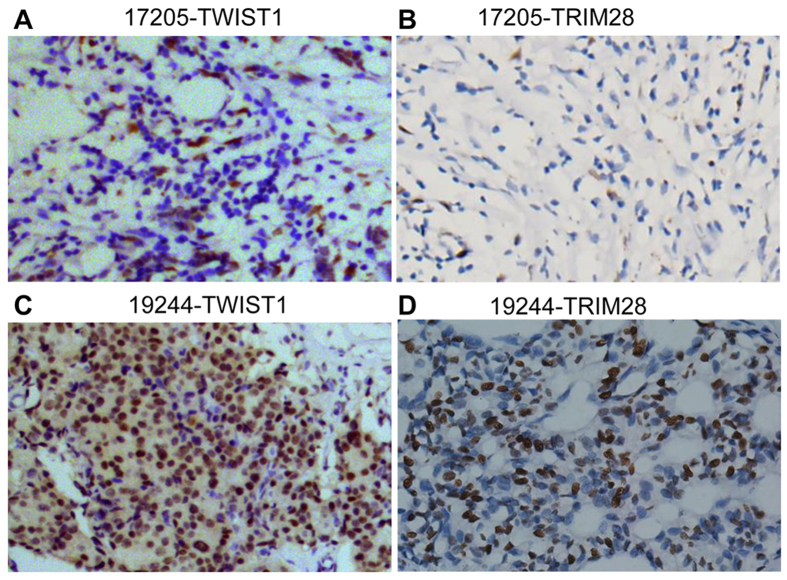
Immunohistochemical (IHC) analysis of TWIST1 and TRIM28 proteins in invasive ductal breast tumors. (**A,B**) Representative IHC of TWIST1 and TRIM28 in the tissue sample No. 17205 respectively. (**C,D**) Representative IHC of TWIST1 and TRIM28 in the tissue sample No. 19244 respectively.

To determine whether TRIM28 plays an important role in breast cancer metastasis, we first compared the mRNA levels of TRIM28 in different breast cancer cell lines (MDA-MB-231, MDA-MB-435, T47D, MCF7 and BT549) with that of non-cancerous epithelial cell line MCF10A. Total RNAs were isolated and qPCR was conducted with primers specifically designed for TRIM28 amplification. As shown in Fig. 1A and compared to the non-cancerous epithelial control (MCF10A), all the cancer cell lines tested showed higher mRNA levels of TRIM28. To determine the correlation between TRIM28 levels and breast cancer development, we collected invasive ductal carcinoma specimens from 33 human breast cancer patients and 11 adjacent normal tissues in Southwest Medical University Affiliated Hospital in China with informed consent. Total RNAs were purified from each tumor tissue and its surrounding normal tissue followed by qPCR. Figure 1B shows that the mRNA levels of TRIM28 in breast cancer tissues were significantly higher (3.5-fold) than that of the adjacent normal tissues. Total proteins were also isolated from each of the cancer samples and their matched adjacent normal tissues and Western blotting was performed using antibodies specifically against TRIM28, TWIST1 or β-actin. As shown in Fig. 1C the protein levels of both TRIM28 and TWIST1 in cancer samples were consistently higher than that of their corresponding adjacent tissues. Of note, most cancer samples expressing higher levels of TRIM28 also showed elevated levels of TWIST1, suggesting a co-relationship between TRIM28 and TWIST1 at their protein levels. To further substantiate the correlation between TRIM28 and TWIST1, immunohistochemistry (IHC) was performed. Again, both TWIST1 and TRIM28 in sample No. 19224 were higher than that of sample No. 17205. In addition, tissues with higher levels of TWIST1 and TRIM28 (Fig. 2C,D) also showed more TWIST1 and TRIM28 positive cells (Fig. 2A,B). Of note, both TWIST1 and TRIM28 were found in the nuclei. These data collectively demonstrate that TRIM28 is not only upregulated in breast cancer cell lines but also positively correlated with the levels of TWIST1 in most invasive breast carcinomas.

### TRIM28 enhances cell migration and invasion

**Figure 3:**
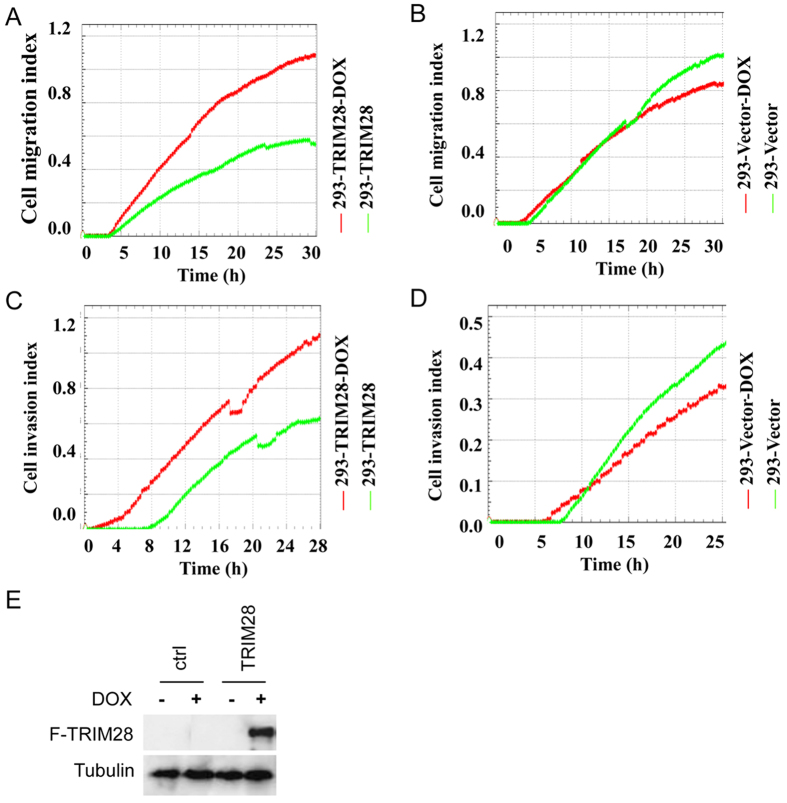
Cell migration (**A,B**), cell invasion (**C,D**) assays with overexpression of TRIM28 or control vector. HEK293-TRIM28 inducible cell line (293-TRIM28) and HEK293-Vector cell line (293-Vector) with or without DOX addition, then cell migration (**A,B**) and invasion (**C,D**) assays. (**E**) Western blotting for Flag tagged vector cell lines (ctrl) and Flag tagged TRIM28 cell lines (TRIM28) without or with DOX addition. The Tubulin served as internal control.

Given the fact that TWIST1 is an important regulator of cell migration and invasion and its expression is highly correlated with the levels of TRIM28 in breast cancers, we decided to explore the effects of TRIM28 on cell migration and invasion. To do so, we established a cell line expressing inducible TRIM28 (293-TRIM28) and a control cell line (293-Vector) with empty vector in HEK293 cells. Cells were treated with doxycycline (DOX) to induce the expression of TRIM28, migration and invasion was monitored with a real time cell analyzer^7^. Figure 3 showed that comparing to that without induction, overexpression of TRIM28 (293-TRIM28 cells treated with DOX) enhanced both migration (Fig. 3A) and invasion (Fig. 3C). However, the effect on migration (Fig. 3B) and invasion (Fig. 3D) were not observed when control cells (293-Vector) were treated with DOX. In fact, both migration and invasion reduced slightly when the control cells were treated with DOX (Fig. 3B,D) and DOX treatment has no effect on cell growth indexes (Data not shown). Figure 3E showed that TRIM28 is expressed in HEK293-TRIM28 but not the control HEK293-Vector cells (293-Vector) when they were treated with DOX.

### TRIM28 upregulates the protein levels of TWIST1

**Figure 4:**
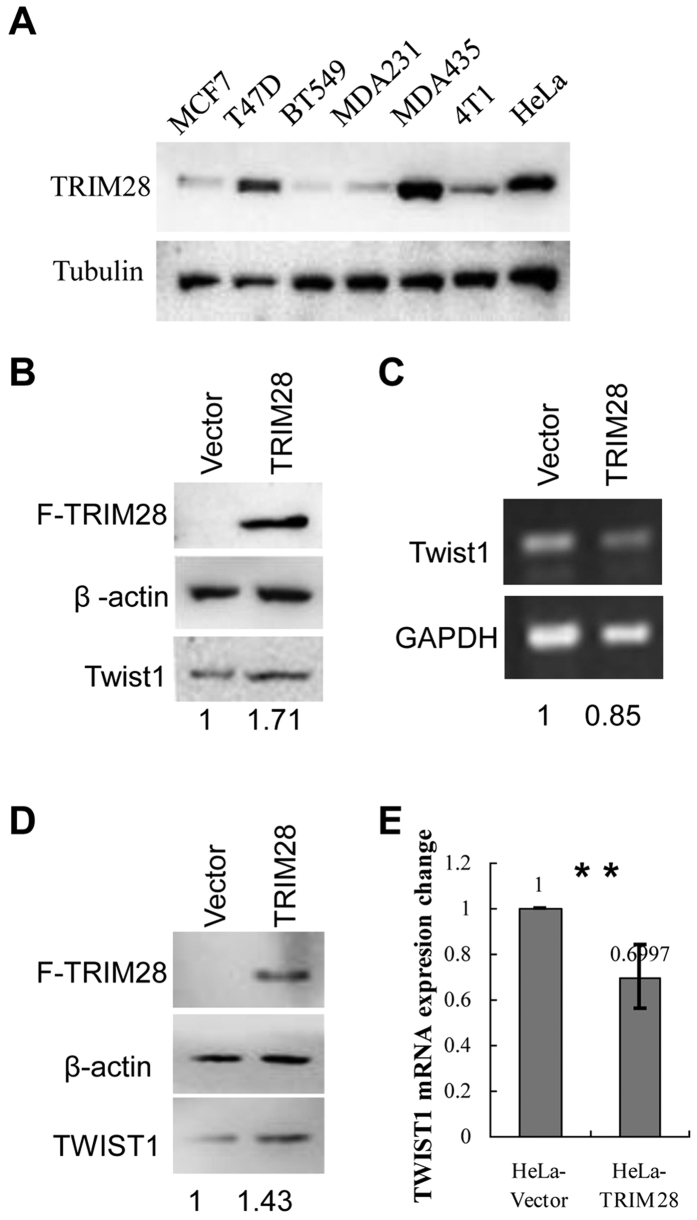
TRIM28 overexpression increases TWIST1 protein levels in 4T1 cells (**B,C**) and in HeLa cells (**D,E**). (**A**) TRIM28 protein expression by Western blotting in MCF7, T47D, BT549, MDA-MB-231(MDA231), MDA-MB-435(MDA435), 4T1 and HeLa cell lines. (**B**) Protein levels by Western blotting in 4T1 cells. (**C**) mRNA levels of TWIST1 in 4T1 cells. TWIST1 protein increases by 1.71 fold but mRNA do not increased (actually decreased) when overexpression of TRIM28. (**D**) TWIST1 protein levels by Western blotting in HeLa cells. (**E**) mRNA levels of TWIST1 by real-time PCR in HeLa cells. TWIST1 protein increases by 1.43 fold but mRNA did not increase (actually decreased) when overexpression of TRIM28 in HeLa cells. **P-value ≤ 0.05.

**Figure 5:**
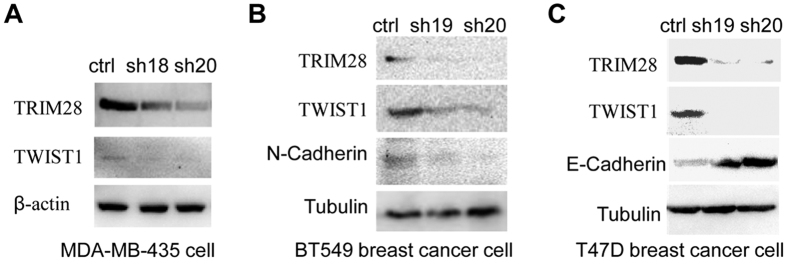
Knockdown of TRIM28 reduces TWIST1 protein level. (**A**) TWIST1 protein expression is reduced when knockdown of TRIM28 in cell line MDA-MB-435. Two plasmids for knockdown of TRIM28, TRIM28-RNAi, Nos: 22918-1 (sh18) and 22920-1 (sh20), respectively, and the non targeted plasmid for control CON007 (ctrl), were transfected into MDA-MB-435 cells for 72 h, Western blotting was performed with indicated antibodies. (**B,C**) TWIST1 protein expression is reduced when knockdown of TRIM28 in cell lines BT549 and T47D respectively. Two plasmids for knockdown of TRIM28, or TRIM28-RNAi, Nos: 22919-1 (sh19) and 22920-1 (sh20), respectively, and one plasmid for non targeted siRNA control CON007 (ctrl), were transfected into cells for 72 h, Western blotting was performed with indicated antibodies.

Since the levels of TRIM28 and TWIST1 are positively correlated in cancer tissues and overexpression of TRIM28 enhances both cell migration and invasion, we want to further determine if TRIM28 functions through TWIST1. To do so, we first conducted a Western blotting assay with the lysates of different cancer cells to identify the lines with high- and low-TRIM28 expression. As shown in Fig. 4A, although TRIM28 is expressed ubiquitously in all cells tested, the levels vary significantly. Comparing to MCF7, BT549, MDA-MB-231 and 4T1 cells, higher levels of TRIM28 are seen in T47D, MDA-MB-435 and HeLa cells. Then we overexpressed TRIM28 in 4T1 mouse breast cancer cells which express low levels of endogenous TRIM28 by transiently transfection of plasmid either expressing Flag-tagged TRIM28 or the empty vector as a control. Two days after transfection, cells were collected and proteins in whole cell lysates (WCLs) were analyzed by Western blotting. As shown in Fig. 4B, the ectopically expressed TRIM28 was detected by antibody against Flag only in cells transfected with expressing plasmid, but not the control. The levels of β-actin in cells with or without TRIM28 overexpression were comparable. However, the protein level of TWIST1 in TRIM28-expressing cells increased significantly (1.7-fold), suggesting that TWIST1 is upregulated by TRIM28. In order to know if TRIM28 affect the mRNA level of Twist1, we conducted semi-quantitative PCR. As shown in Fig. 4C that the mRNA levels of Twist1 were not elevated by TRIM28. In fact, we noticed that the mRNA levels of Twist1 in the TRIM28-overexpressing 4T1 cells appeared to be lower than that of the control. Since this was contradicted with our expectation, we repeated the experiment in HeLa cells and similar results were obtained (Fig. 4D,E). Specifically, the protein level of TWIST1 in HeLa cells ectopically expressing TRIM28 increased significantly (1.43-fold), whereas the mRNA level appeared to be decreased ~30% (p < 0.05). These data collectively suggest that TWIST1 protein is upregulated by TRIM28, but the mRNA levels is unexpectedly down-regulated. To further substantiate these findings, we knocked down TRIM28 in MDA-MB-435 cells, in which higher levels of TRIM28 and TWIST1 were observed, and estimated the levels of TWIST1. As shown in Fig. 5A, TRIM28 was knocked down successfully by both shRNAs (sh18 and sh20) and much lower levels of TWIST1 were seen when TRIM28 was knocked down. Thus, we conclude that TRIM28-mediated TWIST1 upregulation is likely at the post-transcriptional level.

### TRIM28 regulates EMT markers through TWIST1

Down-regulation of cell-cell adhesion molecules E-Cadherin and up-regulation of plastic mesenchymal proteins N-Cadherin are the hallmarks of TWIST1-mediated EMT. We decided to determine if TRIM28 also affects EMT. We knocked down TRIM28 in both T47D and BT549 breast cancer lines and monitored the effect on E-Cadherin and N-Cadherin by western blotting assays. As shown in Fig. 5B,C that TRIM28 is successfully knocked down by shRNAs (sh19 and sh20) in both cell lines. In addition to be consistent with the results mentioned above that knockdown TRIM28 lowered the levels of TWIST1, N-Cadherin and E-cadherin were downregulated and upregulated, respectively, in BT549 and T47 cells when TRIM28 was knocked down (Fig. 5B,C). Of note that no E-Cadherin and N-Cadherin was detected in BT549 cells and T47D cells, respectively. These results not only support the notion that TRIM28 upregulates TWIST1 but also involved in the regulation of the key EMT markers. Nevertheless, the EMT-associated transcriptional factor SNAIL1 was unaffected (data not shown).

### TRIM28 plays important roles in both migration and invasion

**Figure 6:**
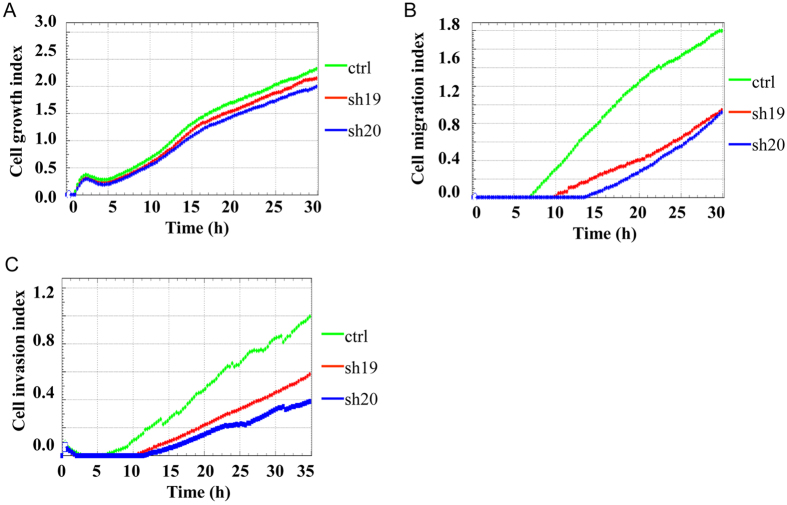
Cell growth (**A**), migration (**B**), and invasion (**C**) assays with depletion of TRIM28 or its control. Two TRIM28 knockdown cell lines (sh19 and sh20) and non targeted siRNA control cell line (ctrl) were knocked down and set up for cell growth, migration and invasion assays in BT549 breast cancer cell line.

Since ectopically overexpressed TRIM28 enhanced migration and invasion and knockdown TRIM28 in BT549 cells affected both TWIST1 protein level and EMT markers, we decided to dissect the role of TRIM28 in cell migration and invasion by knocking down TRIM28 in BT549 cells and monitoring cell movement. Knockdown TRIM28 in BT549 cells reduced cell growth slightly (Fig. 6A), but both cell migration (Fig. 6B) and invasion (Fig. 6C) were repressed dramatically. Together with the fact that TRIM28 overexpression enhanced invasion and migration and TRIM28 plays important roles in the regulation of both TWIST1 and EMT markers, these data suggest that TRIM28 plays an important role in cell migration and invasion by affecting the levels of TWIST1 as well as the EMT processes.

### TRIM28 directly interacts with and stabilizes TWIST1

**Figure 7:**
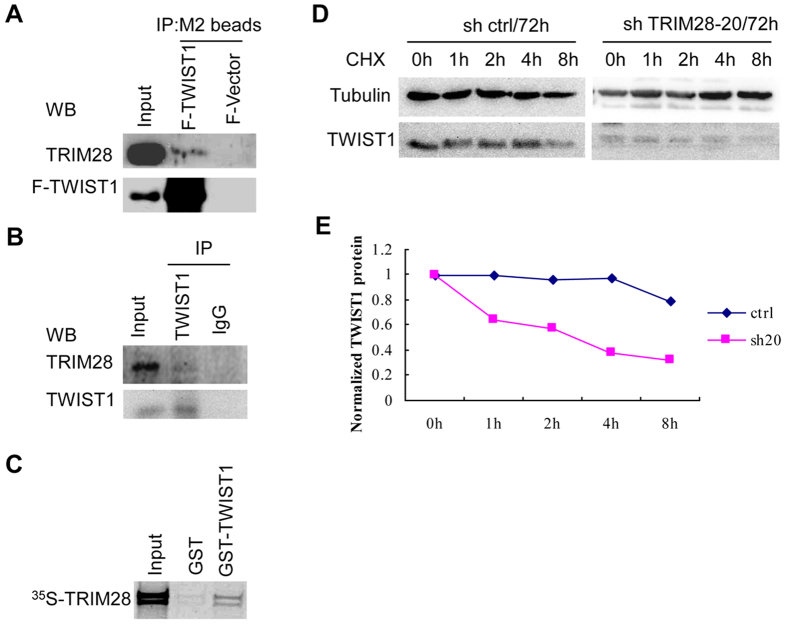
TRIM28 increases TWIST1 stability by interacting with TWIST1 and protecting from degradation. TRIM28 interacts with TWIST1 directly (**A**–**C**). (**A**) Immo-precipitation assay with overexpression. The constructed an inducible HEK293 cell line with over-expressed Flag tagged TWIST1 (F-TWIST1) and vector control (F-Vector), inducible expressed TWIST1 by treating with DOX, IP was performed using M2 beads crosslinked with Flag antibody as described in Materials and Methods. Western blot analysis was performed for determination of protein-protein associations, TRIM28 antibody was used for TRIM28 detection, and Flag antibody was used for TWIST1 overexpression detection. (**B**) Immo-precipitation assay with endogenous TWIST1 by the lysates from 4T1 cells. IP was performed using the lysates from 4T1 cells with TWIST1 antibody and Western blotting with indicated antibodies. (**C**) GST pull-down assay. GST pull-down assay shows the interaction between TWIST1 and TRIM28 directly. Cycloheximide (CHX) chase assay for TWIST1 stability (**D,E**). (**D**) BT549 breast cancer cells were transfected with either non targeted siRNA control (ctrl) or TRIM28-RNAi (sh 20) for 3 days, cells were treated with 0.1 mg/ml of Cycloheximide (CHX) for the indicated hours (h) and Western blotting was performed by indicated antibodies. (**E**) The levels of TWIST1 protein at different time points were quantified when normalized to internal control. Band intensities were semi-quantitatively analyzed by densitometry. The degradation curves were plotted using the time period of CHX treatment for the X-axis and protein band intensities in logarithm for the Y axis.

It has been reported that KAP1 (TRIM28)-dependent stabilization of KRAB-ZNF requires direct interaction^19^. We want to understand if TRIM28-mediated upregulation TWIST1 is also through direct TWIST1 and TRIM28 interaction. To this end, we conducted both immuno-precipitation (IP) and GST pull-down assays. Figure 7A shows that IP with antibody against Flag not only precipitated the flag tagged-TWIST1 but also TRIM28. However, neither of them was precipitated when cells were transfected with empty vector. IP using the lysates from 4T1 cells with antibody against TWIST1 and Western blotting with TRIM28 and TWIST1 further confirmed TRIM28-TWIST1 association (Fig. 7B). Moreover, the results from GST pull-down assays substantiated the fact that TRIM28 directly interacts with TWIST1 (Fig. 7C) and further experiments with truncated TRIM28 found that the C-terminus of TRIM28 interacts with TWIST1 (Supplementary Figure S1A,B). These findings suggest that TRIM28 stabilized TWIST1 indeed by directly interaction.

To explore the mechanisms by which TRIM28 stabilize TWIST1, we monitored the protein levels of TWIST1 when TRIM28 was knocked down in BT549 cells for 72 hours and simultaneously treated with cycloheximide (CHX) with the indicated hour (s) to block protein syntheses (Fig. 7). Representative western blots were shown in Fig. 7D and the intensities of the bands were estimated by Image-J software, normalized with tubulin with the levels of TWIST1 at 0 hour was set as 1. As shown in Fig. 7E, a lower level of TWIST1 is seen in the cells with TRIM28 knocked down (0 hour). When cells were treated with CHX, the protein levels of TWIST1 do not change significantly during the first 4 hours in the control (shCtrl) but reduced dramatically in the cells with TRIM28 knockdown (shTRIM28-20, or sh20). At the endpoint of 8-hour treatment, the levels of TWIST1 decreased by ~21% and ~76% in the control and TRIM28 knockdown cells, respectively. Similar results were obtained with another set of TRIM28 shRNA (sh TRIM28-19, or sh19) (Supplementary Figure S2). Taken together, these data further support the notion that TRIM28 upregulates TWIST1 by inhibiting TWIST1 degradation.

### TRIM28 stabilizes TWIST1 through a mechanism other than affecting TWIST1 ubiquitination

**Figure 8:**
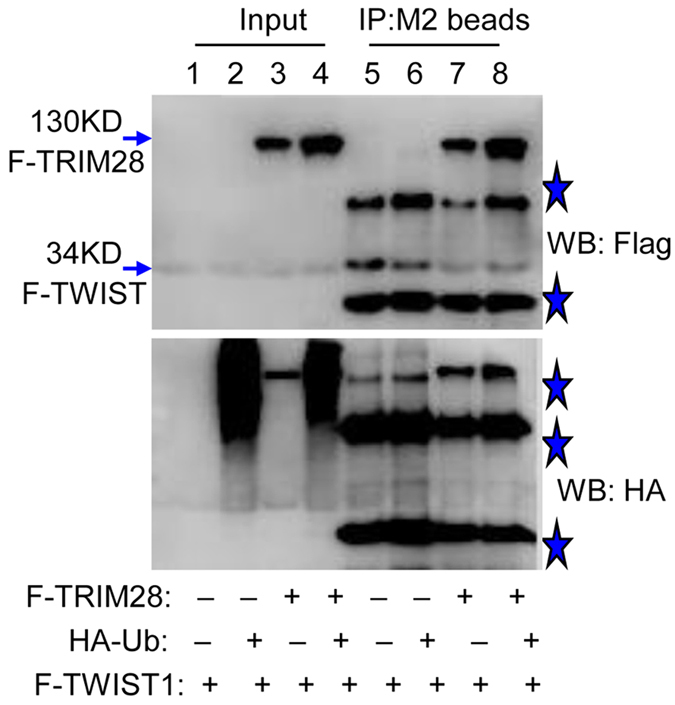
TRIM28 can’t affect on TWIST1 ubiquitination. The *in vivo* ubiquitination assays of TWIST1 were performed by co-transfection of Flag-TWIST1 (F-TWIST1) and either Flag-tagged or untagged TRIM28 in the presence or absence of HA-ubiquitin (HA-Ub) in the HEK 293 cell lines. After IP by Flag antibody (M2 beads), presumed ubiquitinated TWIST1 was detected by HA antibody in Western blotting analysis. The top panel is Western blotting by Flag antibody, and the bottom panel is Western blotting by HA antibody. The stars are non-specific IgG (light and heavy chains of IgG) or an unknown band.

Due to its ability of enhancing p53 ubiquitination, TRIM28 has been suggested to be a protein with ubiquitin E3 ligase activity, albeit MDM2 is the major ubiquitin ligase for p53^18^. We decided to test if TRIM28 stabilizes TWIST1 through affecting the ubiquitination pathway. Flag-tagged TWIST1 was overexpressed in HEK293 cells with or without expression of Flag-TRIM28 and HA-ubiquitin. Immuno-precipitation and Western blotting were conducted. As shown in Fig. 8 that Flag-tagged TRIM28 and TWIST1 (Left of top panel, arrows) and HA-tagged ubiquitin (Left of bottom panel) were successfully expressed in these cells. Both TRIM28 and TWIST1 were precipitated successfully by antibody against Flag (Right of top panel). But TWIST1 appeared to be not ubiquitinated at all (Right of bottom panel). These results suggest that TRIM28 stabilizes TWIST1 through a mechanism other than affecting TWIST1 ubiquitination. However, given the fact that MG132 is capable of inhibiting TWIST1 degradation, we inclined to postulate that the stability of TWIST1 could be partially regulated by ubiquitination pathway (Supplementary Figure S3).

## Discussion

In mammalian cells, TRIM28 is involved in the regulation of dynamic organization of chromatin structure via modification of epigenetic patterns and chromatin compaction. Therefore, it is believed that TRIM28 plays important roles in multiple cellular processes, including gene silencing, cell growth and differentiation, pluripotency, neoplastic transformation, apoptosis, DNA damage response, and maintenance of genomic integrity^14^,^15^,^17^,^25^. Mouse embryos deficient in TRIM28 die before gastrulation, suggesting that TRIM28 plays a pivotal role during embryonic developments^26^,^27^. Emerging evidence also suggests that TRIM28 functions independent of regulation or repression of gene expression instead by serving as a SUMO/ubiquitin E3 ligase or signaling scaffold protein to mediate signal transduction. TRIM28 is capable of binding the E3 ligase MDM2 to promote p53 ubiquitination and degradation^18^. Moreover, it has been reported that TRIM28 itself can serve as a SUMO E3 ligase and by binding tumor suppressor ARF to enhance target protein SUMOylation^28^. Another interesting finding indicates that MAGEA-TRIM28 ubiquitin ligase functions as an oncogene that targets PRKAA1/AMPKα1 for ubiquitination and proteasome-mediated degradation. Degradation of AMPK by MAGEA-TRIM28 results in significantly reduction of autophagy and induction multiple cancer hallmarks^29^. Nevertheless, TRIM28 involves regulation of gene expression at both transcriptional and post-translational levels including ubiquitination/sumoylation. In this study, we found that TRIM28 interacts with and stabilizes TWIST1. We propose that in breast cancer cells exist a TRIM28-TWIST1-EMT axis which plays important roles in breast cancer metastasis.

Increased levels of TRIM28 have been observed in different cancers including liver, gastric, lung, breast, ovarian, pancreatic and prostate cancer, and high levels of TRIM28 usually correlate with a lower survival rate^22^,^30^,^31^,^32^. The tumor suppressive role of TRIM28 is also evident, but mostly in early stages of cancer^33^. In this study, we first showed that comparing to that in the normal adjacent tissues TRIM28 mRNA is significantly higher in breast cancer tissues. In addition, the protein levels of TRIM28 and TWIST1 are not only elevated but also positively correlated with each other, suggesting that TRIM28 and TWIST1 may play important roles in breast cancer development and/or invasiveness. Immunohistochemistry (IHC) further substantiated these findings. To demonstrate that TRIM28 and TWIST1 play important roles in cell migration and invasion, we took the advantage of the inducible system to overexpress TRIM28 in HEK293 cells and knockdown of TRIM28 in BT549 cells. The results showed that overexpression and knockdown of TRIM28 increases and represses migration and invasion, respectively. These findings are in consistent with that reported by Addison *et al.* that TRIM28 is capable of promoting metastatic progression of breast cancer cells *in vitro* and *in vivo*^22^, and both migration and invasion were repressed significantly when TRIM28 is knocked down in BT549 cells.

During EMT, downregulation of cell adhesion molecules E-Cadherin and upregulation of more plastic mesenchymal proteins N-Cadherin and ultimately lead to metastasis. TWIST1 is one of the master regulators of morphogenesis and plays an essential role in EMT and tumor metastasis^1^. We unexpectedly noticed that in 4T1 mouse breast cancer cells ectopically expressed TRIM28 is capable of enhancing the protein level of Twist1 and meanwhile repressing its mRNA level. To determine if this phenomenon is cell-specific, we conducted experiments in HeLa cells and similar results were observed. These findings suggest that TRIM28 may also serve as a general transcriptional corepressor in different cells as suggested by other reports^14^,^15^,^25^,^34^. Nevertheless, TRIM28 is capable of not only upregulating the protein levels of TWIST1 but also regulating EMT markers and ultimately promotes cell migration and invasion.

It has been reported that direct interaction between TRIM28 and KRAB-ZNF is essential for TRIM28-mediated KRAB-ZNF stabilization^22^. We conducted IP and GST pull-down assays and found that TRIM28 is capable of forming a complex with TWIST1. Depleting TRIM28 in BT549 cells reduced the level of TWIST1, suggesting that TRIM28 is a critical factor for TWIST1 stabilization. *In vivo* ubiquitination assays further demonstrated that TRIM28 stabilizes TWIST1 through a mechanism other than the ubiquitination pathway. In fact, TRIM28 is likely to prevent TWIST1 from being ubiquitinated. However, given the fact that MG132 is capable of inhibiting TWIST1 degradation, we inclined to postulate that the stability of TWIST1 could be partially regulated by ubiquitination pathway. Further studies will clarify which E3 ligase(s) involves in TWIST1 ubiquitination and degradation.

The roles of the transcription factor TWIST1 in cancer metastasis have been well established^1^,^2^,^3^,^7^. Identification of the TRIM28-TWIST1-EMT axis in breast cancer cells makes TWIST1 a potential therapeutic target for breast cancer treatment^3^. We recently reported that the natural product Thymoquinone (TQ) can downregulate TWIST1 and inhibit breast cancer cell migration and invasion^32^. It will be interesting to find if TQ is functional through the TRIM28-TWIST1-EMT axis. Finally, multiple lines of evidence showed that TWIST1 is upregulated and/or activated by different upstream factors^3^,^8^,^35^,^36^,^37^,^38^. Therefore, identification of specific factors linking to this axis will not only enhance our understanding the roles of TRIM28 in breast cancer development but also provide more targeting points for breast cancer therapies.

## Materials and Methods

### Ethic statements

Investigation has been conducted in accordance with the ethical standards, the Declaration of Helsinki, national and international guidelines, and has been approved by the Southwest Medical University review board.

### Cell culture

The cell lines MDA-MB-231, MDA-MB-435, T47D, MCF7, BT549, 4T1 and HEK293, were cultured in DMEM or RPMI1640 media (Thermo Fisher Scientific, USA) with 10% fetal bovine serum (FBS) (Hangzhou Sijiqing Biological Engineering Materials Co., Ltd., China)^7^. MCF10A was cultured in DMEM/F12 (1:1) medium (Invitrogen, Cat No: 11330-032) with Horse Serum (Invitrogen, Cat No: 16050-122, 5%), Hydrocortisone (0.5 mg/ml), EGF (20 ng/ml), Cholera Toxin (100 ng/ml), Insulin (10 μg/ml) and Pen/Strep.

### Breast tumor samples, RNA and protein isolation

A total of 33 human breast invasive ductal carcinoma specimens and 11 normal adjacent tissues were collected in Southwest Medical University Affiliated Hospital, China with informed consent^39^. All patients were Chinese women between 33 and 72 years old. Both tumor and adjacent normal tissues were frozen immediately in liquid nitrogen. Total RNA was extracted using RNeasy^®^ mini kit (Cat No: 74104, Qiagen) and stored at −80 °C. For protein extraction, the tissues were homogenized and lysed with ice-cold EBC buffer (20 mM Tris-HCl, pH 8.0, 125 mM NaCl, 2 mM EDTA, and 0.5% NP-40) containing a protease inhibitor cocktail^7^,^40^. Fifty milligrams of proteins were separated in SDS-PAGE and followed by Western blotting assays with indicated antibodies.

### Immunohistochemistry

Immunohistochemistry (IHC) was performed on breast tumor sections prepared from tumor tissues fixed in 10% buffered-formalin and embedded in paraffin as described previously^39^. In brief, sections (4 μm thick) were deparaffinized in xylene and rehydrated in a series of diluted alcohol and distilled water. After antigen retrieval, endogenous peroxidase activity was blocked with 0.3% hydrogen peroxide in methanol for 30 minutes followed by rehydration in 1 × PBS and incubation with 5% rabbit serum for 30 minutes to block nonspecific background. The slides were then incubated overnight at 4 °C with either TWIST1 monoclonal antibody (ab 50887, abcam, USA) or TRIM28 antibody (sc-18146, H-300, Santa Cruz Biotechnology, USA) in Tris-buffered saline containing 2% of serum and 1% of bovine serum albumin (BSA). The washed sections were incubated with appropriate secondary antibodies at dilution of 1:100 for 30 minutes at room temperature followed by peroxidase-conjugated avidin-biotin complexes and 3,3′-diaminobenzidine (DAKO, Glostrup, Denmark). The immunostaining signals were developed by DAB and counterstained with hematoxylin. Sections incubated with TBS containing 2% rabbit serum and 1% BSA without primary antibody served as negative controls.

### Quantitative real time PCR (qPCR)

Cellular and tissue total-RNA was extracted by using RNeasy^®^ mini kit (QIAGEN). RNA concentration was measured by using ND-2000 UV/Vis spectrophotometer (NanoDrop 2000, USA) and final RNA concentrations were set with 150 ng/μl used for cDNA synthesis (reverse transcriptase/RT-PCR). In a 10 μl of RT reaction system, 2 μl of 5 × RT buffer, 1 μl of dNTPs, 0.5 μl of random primer, 0.5 μl of Rev. Ace (enzyme, TOYOBO and BIOBRK companies of China), 0.25 μl of super RI, 0.25 μl of RT-enhancer, 2.25 μl of RNase free water (ddH_2_O) and 3.25 μl of RNA (150 ng/μl) were added. The reaction was completed in a thermocyler (Mastercycler gradient, Eppendorf, Germany) with the following steps: 10 min at 30 °C, 30 min at 42 °C, 5 min at 99 °C. The cDNAs were used as templates for quantitative real time PCR (qPCR). The sequence-specific fluorescence-labeled probes and primers for Taqman qPCR were from the Universal Probe Library Center (Roche, Germany) 18S RNA was used as internal control^32^,^39^,^40^. The information about the primers was presented in Supplementary Table S1. In a 10 μl of the reaction system, 5 μl of 2 × PCR-probe mix, 0.02 μl of probe, 1 μl of primers, 2 μl of H_2_O and 2 μl of cDNA were mixed, and reaction was completed in StepOne plus Thermocycler (Applied Biosystem, Life Technologies, USA) with a 40 cycle of amplification. Relative contents of mRNA were obtained by normalization to 18S RNA and expressed as 2^−ΔΔCT^.

### Western blot analysis

Cells were lysed in EBC buffer^7^,^39^ and 50 μg of proteins were separated on SDS-PAGE and transferred to nitrocellulose membrane (Bio-Rad, USA). The membrane was incubated in 5% milk (1 × TBST) at 4 °C for 2 h followed by incubating in primary antibody solution at 4 °C for 12 h with gentle shaking. The membrane was washed three times with 1 × TBST (1 × TBS plus 0.05% Tween-20) and incubated with secondary antibody tagged with horseradish peroxidase (HRP) for 4~8 h at room temperature with gentle shaking. After another three washes with 1 × TBST (1 × TBSplus 0.05% Tween-20), protein bands were visualized with chemiluminiscence reaction and recorded by the digital imaging system (Universal Hood II, Bio-Rad Lab, Italy)^39^. Antibodies again TWIST1, β-actin, Flag, Tubulin, E-Cadherin, N-Cadherin antibodies have been used previously^7^,^35^,^39^. Antibodies again TRIM28 and Snail1 were purchased from Santa Cruz Biotechnology (sc-33186, H-300) and (sc-10432, E-18), respectively.

### Cell lines with TRIM28-knockdown and overexpression as well as inducible TRIM28

To establish TRIM28 knockdown cell lines, MDA-MB-435 (MDA435), T47D and BT549 breast cancer cells were transfected with shRNA either targeting TRIM28 (Nos: 22917-1, 22918-1, 22919-1 and 22920-1, respectively) negative control CON007 (Shanghai Genechem Co., Ltd., Shanghai, PRC). About 900 ng of shRNA plasmids were transfected into the cells at 30–40% confluence, and 72 h post transfection whole cell lysats (WCLs) were prepared. For TRIM28 overexpression, 900 ng of pcDNA5/FRT/TO-TRIM28 plasmid or pcDNA5/FRT/TO vector was transfected into 4T1 or HeLa cells at 40–50% confluence and WCLs were made after 36–48 h transfection. To establish the cell line (293-TRIM28) with inducible expression of TRIM28, recombinant plasmid pcDNA5/FRT/TO-TRIM28 was constructed and transfected into Flp-In™T-REx™-HEK293 cells. TRIM28 expression was induced by Doxcycline (DOX) at a concentration of 0.1 μg/ml^41^. The HEK293 cells transfected with pcDNA5/FRT/TO empty vector (293-Vector) serves as negative controls^7^. Cell migration, invasion and growth index were estimated using a real time cell analyzer (xCELLigence RTCA DP, Roche, Germany)^7^.

### Cell migration and invasion assays

To measure cell growth index, 100 μl of cell suspensions (5 × 10^4^ cells/ml) were seeded in each of the 16 well E-plate. CMI plates were used for cell migration and invasion index analysis with the lower chambers filled with chemotaxis inducer (10% serum supplemented media) and upper chamber filled with 100 μl of cell suspensions (5 × 10^4^ cells/ml) in serum free medium. For cell invasion assay, the membrane of the CMI plate was coated with Matrigel (Catalogue number 354277, BD Biosciences, USA) diluted in 1 × PBS buffer (1:40) before the cells were seeded. Cell migration and invasion status were checked every 30 min. All experiments were done in a real time cell analyzer (xCELLigence RTCA DP, Roche, Germany)^7^,^35^,^39^,^42^ and all assays were conducted at least two times.

### Immunoprecipitation and Western blotting

Immunoprecipitation (IP)-Western blotting analyses were as described previously^7^,^42^. For IP-Western analysis using WCLs, Control or HEK293 cells expressing Flag-tagged TWIST1^7^ were washed twice in PBS and lysed in ice-cold EBC buffer (20 mM Tris-HCl, pH 8.0, 125 mM NaCl, 2 mM EDTA, and 0.5% NP-40) with protease inhibitors. The lysates were centrifuged at 13,000 rpm for 15 min at 4 °C to remove cell debris. The whole-cell extracts were precleared with protein A/G-conjugated Sepharose beads for 2 h at 4 °C with gentle agitation. After a high-speed centrifugation to remove beads, the supernatants were incubated with anti-Flag M2 monoclonal antibody attached on agarose beads (ZEview^**TM**^ Red ANTI-FLAG M2 Affinity Gel, F2426, Sigma, USA) for 3 h at 4 °C with rotation. Beads were collected and washed extensively with immunoprecipitation washing buffer^7^. The precipated proteins were resolved on 10% SDS-PAGE and Western blot was done antibodies against Flag (F3165, Sigma, USA) or other proteins.

### GST pull-down assay

GST or GST-TWIST1 fusion protein was expressed in BL21 *E. coli* and purified with glutathione-sepharose 4B breads (Amersham Pharmacia Biotech). ^32^S-labeled TRIM28 protein was synthesized using the TNT *in vitro* transcription-translation kit (Promega). The Pull-down assays were carried out as described^7^,^42^. In brief, the beads with GST or GST-TWIST1 fusion protein were incubated with ^35^S-labeled TRIM28 for 2 h at 4 °C, washed five times with washing buffer (20 mM Tris-HCl, 150 mM NaCl, 1 mM dithiothreitol, 1 mM EDTA, 0.1% NP-40 with protease inhibitors). The proteins on the beads were used for Western blotting assays.

### Ubiquitination assay

*In vivo* ubiquitination assays were performed in HEK 293 cells co-transfected with plasmids expressing Flag-TWIST1 and Flag-tagged or untagged TRIM28 in the presence or absence of HA-ubiquitin (HA-Ub)^43^. Proteins were immune-precipitated with antibody against Flag attached on M2 beads (ZEview^**TM**^ Red ANTI-FLAG M2 Affinity Gel, F2426, Sigma) and used for Western blotting with antibody against HA.

### Cycloheximide-based protein stability assay

BT59 breast cancer cells were transfected with plasmids expressing either siRNA control (CON007, ctrl) or siRNA against TRIM28 (TRIM28-RNAi, 22919-1, sh19) (Shanghai Genechem Co., Ltd., Shanghai, PRC) and cultured for 64 h before 0.1 mg/ml of Cycloheximide (CHX) (Sigma, USA) was added. Cells were harvested and proteins in lysates were used for Western blotting. Band intensities were semi-quantified by densitometry and analyzed using Adobe photoshop CS3 software.

### Statistical analysis

Results were pooled from three separate experiments. Paired Student’s t-test was used to determine any significant differences. *P*-value ≤ 0.05 was considered as a significant difference.

## Additional Information

**How to cite this article**: Wei, C. *et al.* Tripartite motif containing 28 (TRIM28) promotes breast cancer metastasis by stabilizing TWIST1 protein. *Sci. Rep.* **6**, 29822; doi: 10.1038/srep29822 (2016).

## Supplementary Material

Supplementary Information
