# Supplementary material for: Tripartite motif containing 28 (TRIM28) promotes breast cancer metastasis by stabilizing TWIST1 protein
Source: Sci Rep. 2016 Jul 14;6:29822. doi: 10.1038/srep29822 (PMC4944148; doi:10.1038/srep29822)
Supplement: Supplementary Information [file srep29822-s1.pdf]

Tripartite motif containing 28 (TRIM28) promotes breast cancer metastasis by stabilizing TWIST1 protein

Chunli Wei, Jingliang Cheng, Boxv Zhou, Li Zhu, Md. Asaduzzaman Khan, Tao He, Sufang Zhou, Jian He, Xiaoling Lu, Hanchun Chen, Dianzheng Zhang, Yongxiang Zhao, Junjiang Fu

Supplementary Figure S1

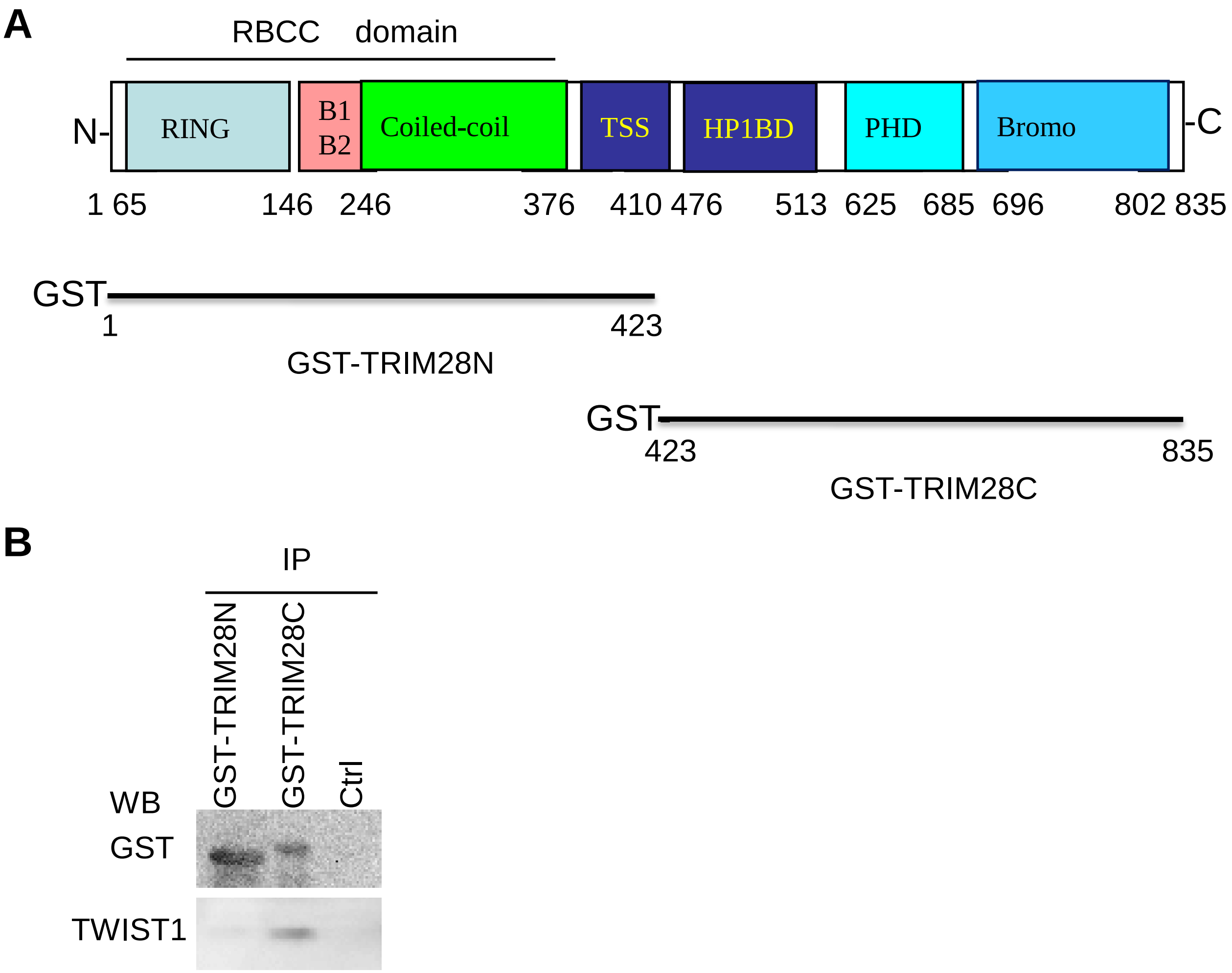

Supplementary Figure S1. The C-terminus of TRIM28 interacts with TWIST1. A. The structure of GST fused C and N terminus of TRIM28. The sizes and positions of truncated TRIM28 were indicated. B. GST pull-down assay was performed using purified GST fused TRIM28 different proteins and IP with lysates from BT549 cells, respectively, and Western blotting with indicated antibodies. TWIST1 monoclonal antibody was purchased from Abcam (ab 50887, abcam, USA), GST rabbit polyclonal antibody was purchased from Santa Cruz Biotechnology (sc-33613, USA).

Supplementary Figure S2

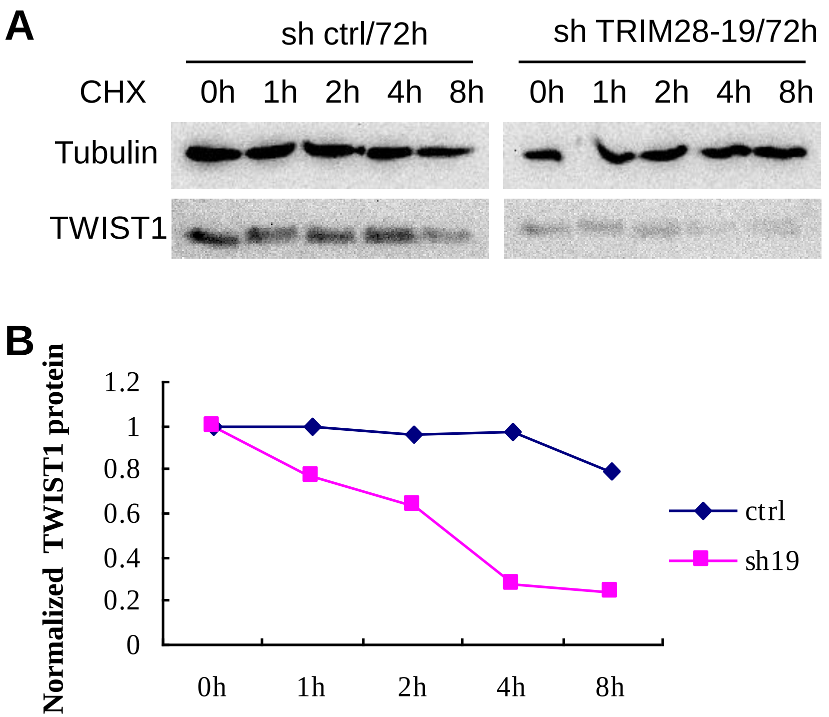

Supplementary Figure S2. Cycloheximide (CHX) chase assay for TWIST1 stability. A, BT549 breast cancer cells were transfected with either non targeted siRNA control (ctrl) or TRIM28-RNAi (sh 19) for 3 days, cells were treated with 0.1 mg/ml of Cycloheximide (CHX) for the indicated hours (h) and Western blotting was performed by indicated antibodies. B. The levels of TWIST1 protein at different time points were quantified from A when normalized to internal control. Band intensities were semi-quantitatively analyzed by densitometry. The degradation curves were plotted using the time period of CHX treatment for the X-axis and protein band intensities in logarithm for the Y axis.

Supplementary Figure S3

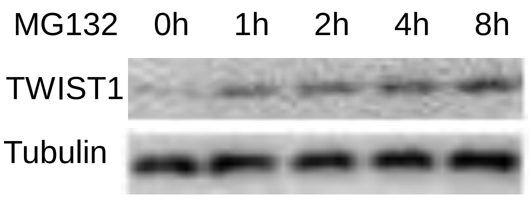

Supplementary Figure S3. MG132 treatment stabilizes TWIST1 protein. MDA-MB-435 cancer cells were treated with 20μM of MG132 for the indicated hours (h) and Western blotting was performed with indicated antibodies.

Supplementary Table S1. Primer sequences for q-RT-PCR (qPCR) or Semi-RT-PCR (Semi-PCR) used for Human and Mouse

Name	Sources	Sequence (5'-3')	Usage	probe
Q18S-48L	Both	gcaattattccccatgaacg	qPCR	48
Q18S-48R	Both	gggacttaatcaacgcaagc	qPCR	48
TWIST1-6L	Human	ggcatcactatggactttctctatt	qPCR	6
TWIST1-6R	Human	ggccagtttgatcccagtatt	qPCR	6
TRIM28L49	Human	atggtgcagacagcactgg	qPCR	49
TRIM28R49	Human	gcagtacacgctcacatttcc	qPCR	49
Twist1-58L	Mouse	agctacgccttctccgtct	qPCR	58
Twist1-58R	Mouse	tccttctctggaaacaatgaca	qPCR	58
Twist1-5	Mouse	ggccaggtacatcgacttcc	Semi-PCR	
Twist1-3	Mouse	ggcctgtctcgctttctctt	Semi-PCR	
GAPDH-5	Mouse	acagtcagccgcatcttctt	Semi-PCR	
GAPDH-3	Mouse	ttgattttggagggatctcg	Semi-PCR	
